# Development of a prediction model for clinically-relevant fatigue: a multi-cancer approach

**DOI:** 10.1007/s11136-024-03807-9

**Published:** 2024-11-09

**Authors:** Dhirendra Adiprakoso, Dimitris Katsimpokis, Simone Oerlemans, Nicole P. M. Ezendam, Marissa C. van Maaren, Janine A. van Til, Thijs G. W. van der Heijden, Floortje Mols, Katja K. H. Aben, Geraldine R. Vink, Miriam Koopman, Lonneke V. van de Poll-Franse, Belle H. de Rooij

**Affiliations:** 1 Department of Health Technology and Services Research (HTSR), Technical Medical Centre, University of Twente, Enschede, The Netherlands; 2 Department of Research and Development, Netherlands Comprehensive Cancer Organisation (IKNL), Utrecht, The Netherlands; 3 CoRPS-Center of Research On Psychological Disorders and Somatic Diseases, Department of Medical and Clinical Psychology, Tilburg University, Tilburg, The Netherlands; 4 Department of Medical Oncology, University Medical Center Utrecht, Utrecht University, Utrecht, The Netherlands; 5 Division of Psychosocial Research & Epidemiology, The Netherlands Cancer Institute, Amsterdam, Netherlands; 6 Department of IQ Health, Radboud University Medical Centre, Nijmegen, The Netherlands

**Keywords:** Prediction modelling, Machine-learning, Cancer-related fatigue, Clinically relevant fatigue, Health-related quality of life, Cancer survivors

## Abstract

**Purpose:**

Fatigue is the most prevalent symptom across cancer types. To support clinicians in providing fatigue-related supportive care, this study aims to develop and compare models predicting clinically relevant fatigue (CRF) occurring between two and three years after diagnosis, and to assess the validity of the best-performing model across diverse cancer populations.

**Methods:**

Patients with non-metastatic bladder, colorectal, endometrial, ovarian, or prostate cancer who completed a questionnaire within three months after diagnosis and a subsequent questionnaire between two and three years thereafter, were included. Predictor variables included clinical, socio-demographic, and patient-reported variables. The outcome was CRF (EORTC QLQC30 fatigue ≥ 39). Logistic regression using LASSO selection was compared to more advanced Machine Learning (ML) based models, including Extreme gradient boosting (XGBoost), support vector machines (SVM), and artificial neural networks (ANN). Internal–external cross-validation was conducted on the best-performing model.

**Results:**

3160 patients were included. The logistic regression model had the highest C-statistic (0.77) and balanced accuracy (0.65), both indicating good discrimination between patients with and without CRF. However, sensitivity was low across all models (0.22–0.37). Following internal–external validation, performance across cancer types was consistent (C-statistics 0.73–0.82).

**Conclusion:**

Although the models’ discrimination was good, the low balanced accuracy and poor calibration in the presence of CRF indicates a relatively high likelihood of underdiagnosis of future CRF. Yet, the clinical applicability of the model remains uncertain. The logistic regression performed better than the ML-based models and was robust across cohorts, suggesting an advantage of simpler models to predict CRF.

**Supplementary Information:**

The online version contains supplementary material available at 10.1007/s11136-024-03807-9.

## Introduction

Globally, the cancer survival rate has increased over time as standards for treatment and care continually improve [1]. However, patients with cancer continue to experience long-term consequences [2], with fatigue being the most prevalent across cancer types, cancer treatment regimens, and throughout different stages of the disease trajectory [3]. Furthermore, across a wide range of cancer types, patients often experience persistent fatigue after treatment [2, 4, 5], which can have a long-term negative effect on a patient’s health-related quality of life (HRQoL) [2, 4, 6]. Hence, patients might benefit from early fatigue-related supportive care [4].

A common method to measure a patient’s fatigue is by using patient reported outcome measures (PROMs). The European Organisation for Research and Treatment of Cancer Quality of Life Questionnaire Core-30 (EORTC QLQ-C30) [7], which assesses patients functioning and symptoms, has been used and validated in various cancer populations [8, 9]. Clinical relevance thresholds have been defined for the EORTC QLQ-C30 to improve the interpretation of the scores in daily clinical practice [10]. Using this threshold, predicting future clinically relevant fatigue (CRF) would support clinicians in selecting patients who need long-term fatigue-related supportive care.

To better predict CRF in patients with cancer, prediction models can be defined and used to anticipate a patients’ future level of fatigue to inform patients and their health care team of their potential need for supportive care [11]. However, prediction models for cancer are more commonly used to predict cancer survival [12–14], support diagnostics, and, to a lesser extent, provide information on HRQoL [15, 16]. Within predictive modelling, machine learning (ML) is increasingly utilized [17–19]. To date, few ML-based models have been developed specifically to predict cancer-related fatigue. These models were predominantly cancer-specific, such as for breast cancer [6, 18]. Such specification overlooks a wider adoption of CRF-related prediction across cancer types [6]. Further, the performance of previously reported models predicting CRF varied across studies due to missing relevant predictors, such as functioning and symptoms [19], or having homogeneous data [18]. Therefore, developing a ML-based prediction model validated for patients with multiple cancer types using PROMs as predictors would address the research gaps currently present within the cancer-related fatigue domain.

The aim of this study is to develop and compare prediction models to predict CRF in multiple, heterogeneous cohorts of patients with non-metastatic cancer between two and three years after diagnosis [20], using ML-based models. Predictors include clinical and sociodemographic factors, as well as HRQoL domains (functioning and symptoms) reported within three months after diagnosis. In addition, the study aims to assess the validity of such prediction models across diverse cancer populations.

## Methods

### Data collection

In this study, data were derived from the Patient-Reported Outcomes Following Initial Treatment and Long-term Evaluation of Survivorship (PROFILES) registry [21, 22]. PROFILES contains longitudinal sociodemographic and EORTC QLQ-C30 data from various cohort studies of patients diagnosed with different cancer types. Data from the Netherlands Cancer Registry (including patient-, treatment-and tumour-related characteristics) were linked with patient-reported outcomes from the PROFILES registry, using a linkage key (if available), or based on personal identifiers (name(s) and date of birth) [22]. This study used data from cohort studies of patients with non-metastatic cancer at diagnosis (TNM stage I-III), including colorectal cancer (Prospective Dutch Colorectal Cancer [PLCRC] cohort [23] and Patient-Reported Outcomes in Colorectal Cancer [PROCORE]), gynaecological cancer (Registration System Oncological Gynaecology [ROGY] care trial [24]), bladder cancer (Insight into bladder cancer care [BlaZIB] [25]), and prostate cancer (Insight into prostate cancer care [ProZIB] [26]). All available cohort studies were included, as cancer type was not considered an in- or exclusion criterion.

Questionnaires were completed between July 2013 and October 2017 for PLCRC, between January 2016 and January 2019 for PROCORE, between April 2011 and March 2014 for ROGY, between November 2017 and October 2019 for BlaZIB, and between October 2015 and April 2016 for ProZIB. For each cohort study, data were collected using different time intervals between follow-ups (Appendix 1). Patients were included if they completed a questionnaire within three months since cancer diagnosis (T_baseline_), and at least one subsequent questionnaire between two and three years after diagnosis (T_endpoint_). Patients who did not complete the fatigue sub-scale of the EORTC QLQ-C30 were excluded. Unrealistic datapoints (e.g., BMI above 105.7 and below 6.7) were set to missing (Appendix 2). All preparation and pre-processing steps were done using the “*dplyr*” package version 1.1.0 in R version 4.2.1 [27, 28].

## Measures

### Predictors

Sociodemographic predictor variables obtained from the questionnaire at T_baseline_ included the patient’s age at completion of the first questionnaire, highest level of education (“lower/primary education”, “secondary education (high school)”, “secondary vocational education”, or “vocational education/university”), marital status (“married/cohabitating” or “single/divorced”), smoking status (“never smoker”, “previous smoker”, or “current smoker”), current alcohol use (“no” or “yes”), sex (“male” or “female”), BMI (kg/m^2^), and the sum of comorbidities reported using a modified version of the Charlson Index [29] was categorized (“none”, “one”, or “two or more”). Comorbidities were self-reported and included whether in the previous 12 months, patients had heart disease, stroke, hypertension, asthma, chronic bronchitis, COPD, diabetes, peptic ulcer, kidney disease, liver disease, blood disease, thyroid disease, depression, osteoarthritis, back pain, and/or rheumatoid arthritis.

Clinical predictor variables were obtained from the NCR and included cancer type (“ovarian cancer”, “endometrial cancer”, “colorectal cancer”, “bladder cancer”, or “prostate cancer”), TNM cancer stage for colorectal, bladder, and prostate cancer, and FIGO stage for ovarian and endometrial cancer (“I”, “II”, or “III”), time since diagnosis (in days), and time between diagnosis and start of treatment (in days). Treatment-related predictor variables were dichotomised (“no” or “yes”) and included whether a patient received surgery, radiotherapy, or systemic treatment (i.e., chemotherapy, targeted therapy, or immunotherapy) at T_baseline_. Cancer-specific treatment-related variables were omitted due to overlap with cancer type.

Patient-reported outcome (PRO) predictor variables included the functioning and symptom scores derived from the EORTC QLQ-C30 questionnaire version 3.0, including global quality of life, functioning scales (physical functioning, role functioning, emotional functioning, cognitive functioning, social functioning) and symptom scales (fatigue, nausea/vomiting, pain, dyspnoea, sleeping disturbance, appetite loss, constipation, diarrhoea, and financial difficulties) at T_baseline_. Answers on the functioning and symptom scales were reported on a 4-point Likert scale (“Not at all”, “A bit”, “Quite a bit”, and “Very much”) and transformed to linear scores ranging from 0 to 100 scale, with higher scores indicating better functioning or more symptoms [7].

### Outcome

The models’ outcome was CRF at T_endpoint,_ as measured with the three items of the EORTC QLQ-C30 fatigue scale (“Did you need to rest?”, “Have you felt weak?” and “Were you tired?”). The outcome was dichotomised based on clinical thresholds defined by Giesinger et al. [10]. For fatigue, the cut-off point was set at 39, meaning that scores of 40–100 indicated the presence of CRF [10]. For sensitivity analysis, different thresholds were applied to the best performing model (i.e., 20, 30, 50, and 60).

### Multiple imputation

For EORTC QLQ-C30 variables at T_baseline_, scoring guidelines set by Aaronson et al. [7] were followed. After examining the collected data, missing datapoints were found to be due to patients not completing the questionnaires fully at both T_baseline_ and at T_endpoint_. If only complete cases were used, this would result in an information loss of over 70%. Moreover, a patients’ data at T_baseline_ could not be copied over to T_endpoint_ or vice-versa, as this might risk an unrealistic representation of the patient. The data were considered to be missing at random. Therefore, missing data for all predictor variables at T_baseline_ were imputed to avoid further information loss. Since the missing data were deemed to be missing at random, multiple imputation was performed [30]. Multiple imputation was conducted using the “MissRanger*”* package (version 2.2.1) [30, 31]. The ‘MissRanger’ method is a non-parametric imputation method that can handle diverse forms of input data [31–33]. Hyperparameters tuned for imputation included the number of iterations and number of trees. The default output of 10 imputation datasets was used.

## Model development

Alongside logistic regression, eXtreme Gradient Boosting (XGBoost), support vector machines (SVM), and artificial neural networks (ANN) were employed in this study. Each algorithm had demonstrated good results as a tool for developing prediction models in previous studies and reviews [12, 13, 20, 33–35]. All model development steps (Appendix 3) were conducted using various packages under R version 4.2.1 [27].

First, a randomly selected 80% of the data was used for model development and the remaining 20% was used for model testing. Categorical predictor variables were modelled using one-hot encoding [36]. Hyperparameter tuning was performed using a random search, validated through ten-fold cross-validation, and used balanced accuracy as the defining criterion. Class imbalance, where the ratio between the number of patients with CRF (minority class) and the number of patients without (majority class) was too high to reliably develop a prediction model, was addressed using the Synthetic Minority Oversampling Technique (SMOTE). SMOTE utilizes k-Nearest Neighbours to create synthetic examples of the minority class in the outcome measure, thereby oversampling the minority class [37]. This study used the default k = 5 neighbours, and the default ratio between the minority and majority class was 1:3, oversampling the minority class such that 50% of the model development data consisted of synthetic examples.

### Logistic regression

In the logistic regression model, predictor variables were selected using the Least Absolute Shrinkage and Selection Operator method (LASSO). This method selects predictor variables by attempting to set the variables’ respective regression coefficients to zero using a pre-defined constraint (alpha) [37]. We did not add interaction variables because they would make the logistic model too complex and unexplainable for clinical use, and because the more advanced models described below capture interaction effects by their architecture. Predictor variables were checked for collinearity. The “*glmnet*” package version 4.1–6 [38] was used for model building and validation.

### eXtreme gradient-boosting (XGBoost)

XGBoost is an ML algorithm that is a more scalable form of decision tree boosting, iteratively combining decision trees while utilising a weighted quantile sketch to approximate tree learning [35]. For classification problems, XGBoost can learn a full tree using a greedy approach more rapidly compared to simpler tree boosting methods [35]. For this study, the hyperparameters tuned included the number of boosting iterations, maximum tree depth, learning rate, minimum loss reduction, subsample ratio for each tree, minimum weight of new leaves, and subsample percentage. The “*xgboost*” package version 1.7.3.1 [39] was employed for model building and validation.

### Support vector machine (SVM)

SVM maps input data using a pre-defined function and fits the data using a structural risk minimisation (SRM) principle [40]. The SRM principle is used to minimise the generalisation error of a model, thereby aiding the generalisability of the model [41]. For this study, the cost constant, “C”, was the hyperparameter used for tuning. The “*e107c1*” package version 1.7–13 [42] was used for model building and validation.

### Artificial neural network (ANN)

ANN is a computational system that resembles the human brain, whereby neurons are used to receive, transfer, and interpret information [43]. A simple feedforward structure with the number of hidden layers defined during hyperparameter tuning was employed in this study. The “*nnet*” package version 7.3–17 [44] was utilized for model building and validation.

### Statistical analysis and calibration

Following multiple imputation, each model was applied on each imputed dataset. Results were pooled by averaging the output from each imputed dataset. Baseline characteristics were reported for each cohort as an average from all imputed datasets using means and standard deviations for normally distributed continuous variables, medians and interquartile ranges for non-normally distributed variables, and frequencies and percentages for categorical variables. The univariable associations between predictor variables and the outcome measure (dichotomised CRF at T_endpoint_) were assessed using the Wilcoxon rank-sum test for non-normally distributed continuous predictors (based on Shapiro–Wilk and Kolmogorov–Smirnov tests) and chi-square test for categorical predictors. P-values below 0.05 were considered statistically significant.

Statistical analyses included a comparison of the accuracy, balanced accuracy, precision, sensitivity, specificity, and F1-score of each model on the test set. Bootstrapping over 1000 iterations was used to estimate the stability of each models’ predictions [45]. In addition, 95% confidence intervals over the bootstrap iterations were reported. For this study, a higher score on each statistical measure indicated a better-performing model, with scores at and above 0.8 considered indicative of good statistical performance. While accuracy, precision, and F1-score measure how well a model makes correct predictions, sensitivity and specificity reflect a model’s discriminative ability [46, 47]. Balanced accuracy avoids potential bias due to class imbalance [48] by averaging the accuracy between outcome measure classes (with/without CRF at T_endpoint_). For sensitivity analysis, each performance measure was calculated per fatigue threshold.

Following statistical analyses, receiver operating curves (ROC) were plotted, and the area underneath the curve (AUC-statistic), also referred to as the C-statistic, was calculated on the test set [16], in which a higher score indicating a better performing model. Bootstrapping over 1000 iterations was used to validate the C-statistics of each prediction model, with 95% confidence intervals. The same steps were applied for sensitivity analysis per threshold.

Calibration on the test set was conducted to gain additional insight into how each model’s result compared to the predicted and observed outcomes [49]. First, calibration plots were created to identify patterns of miscalibration, with the calibration line and its confidence interval plotted alongside an ideal. This indicated whether the model was valid in specific subgroups based on predicted outcomes [50]. The calibration plot’s slope and R-squared value were calculated and compared between each model. Both performance measures indicated the extent of a model’s calibration, showing over-/underfitting [50, 51].

### Internal–external cross-validation

Further analyses were conducted to explore the robustness and generalisability of the final model across the five cohorts included in the model. The internal–external cross-validation approach proposed by Steyerberg and Harrell [45] was chosen since it provided the most optimal and stable manner of validating models that include multiple heterogeneous cohorts. Recent studies have highlight the importance of internal–external validation for prediction heterogeneity [45, 52]. Internal–external cross-validation utilises a similar approach to *k-*fold cross-validation, where instead of splitting the model development data into folds, each cohort is treated as a fold. Each cohort-based fold is then iteratively used to validate and statistically analyse a model trained using the remaining cohorts. Thus, each cohort is treated similarly, leading to a final model that fully incorporates the nuances of each cohort and their cancer type. Finally, a meta-analysis was conducted on the C-statistics to understand the performance difference between each fold. A forest plot was used to visualise the extent of this difference. Sensitivity analysis was conducted through a meta-analysis based on cancer type.

## Results

### Patient characteristics

**Fig. 1:**
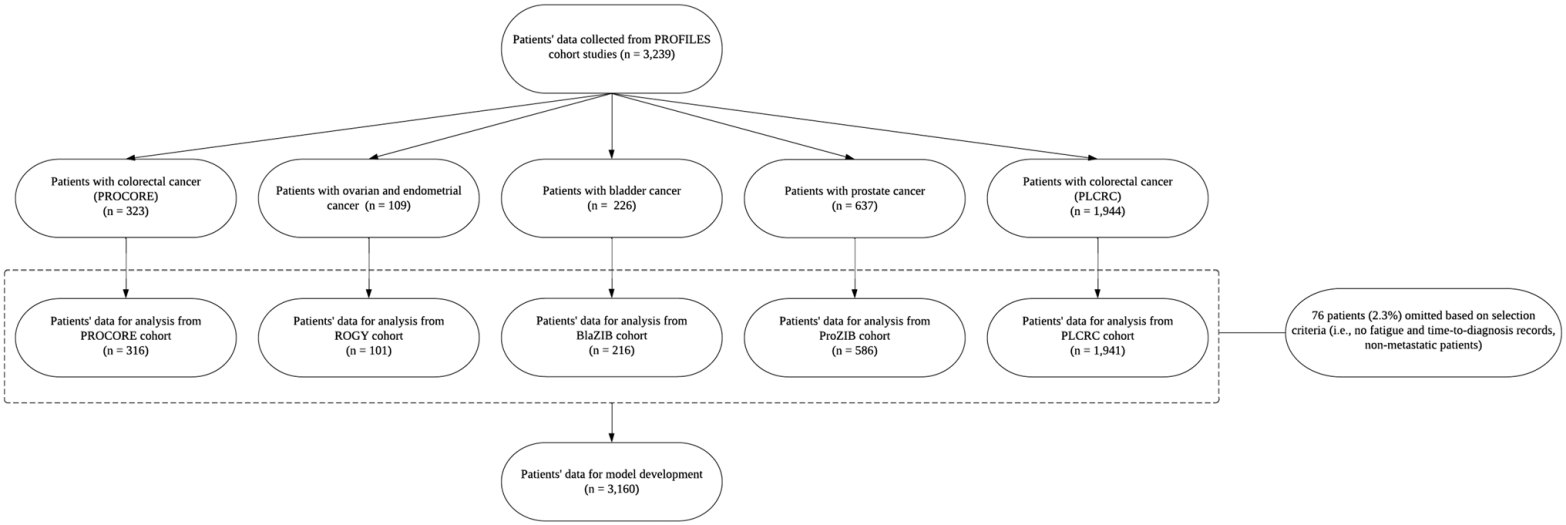
Patient data collection journey

Outcome data from 3160 patients were used for model development (Fig. 1). At T_baseline_, most patients had completed vocational/higher education (N = 1155; 36.6%), were married (N = 2485; 78.6%), had a history of smoking (N = 1807; 57.2%), were currently drinking alcohol (N = 1614; 51.1%), had two or more comorbidities (N = 1127; 35.7%), and had undergone surgery (N = 2527; 80.0%). Among the cohorts with male patients than female, the colorectal and bladder cancer cohorts had more male patients than female (PLCRC: N = 1234 (63.6%) vs 707 (36.4%), PROCORE: N = 196 (62%) vs 120 (38%), and bladder cancer cohort: N = 173 (80.1%) vs 43 (19.9%)) (Table 1). At T_baseline_, the colorectal cancer cohort had more patients with stage III cancer, while the gynaecological, prostate, and bladder cancer cohorts had more patients with TNM or FIGO stage I cancer. Moreover, within each cohort at T_baseline_, the median age ranged between 65 and 70 years ((colorectal cancer cohorts: PROCORE: 67.0 (IQR = 14.0), PLCRC: 66.1 (IQR = 13.1), gynaecological cancer cohort: 65.0 (IQR = 13.0), prostate cancer cohort: 69.0 (IQR = 9.0), bladder cancer cohort: 70 (IQR = 11.0)) (Table 1).

**Table 1:** Sociodemographic and clinical characteristics and patient-reported outcomes at T_baseline_

Variable	Colorectal cancer—PROCORE (N = 316; N%)	Gynaecological cancer (N = 101; N%)	Prostate cancer (N = 586; N%)	Colorectal cancer—PLCRC (N = 1941; N%)	Bladder cancer (N = 216; N%)
Age at questionnaire, median (IQR)	67.00 (14.00)	65.00 (13.00)	69.00 (9.00)	66.06 (13.06)	70.00 (11.00)
Education level					
Lower/Primary	29 (9.2)	9 (8.9)	28 (4.8)	89 (4.6)	19 (8.8)
Secondary (High school)	70 (22.2)	81 (80.2)	192 (32.8)	628 (32.4)	42 (19.4)
Secondary (Vocational)	129 (40.8)	11 (10.9)	156 (26.6)	432 (22.3)	90 (41.7)
Vocational/University	88 (27.8)	0 (0.0)	210 (35.8)	792 (40.8)	65 (30.1)
Marital status, N (%)					
Married/ Cohabitating	264 (83.5)	84 (83.2)	525 (89.6)	1,609 (82.9)	176 (81.5)
Single/Divorced	52 (16.5)	17 (16.8)	61 (10.4)	332 (17.1)	40 (18.5)
Smoking history, N (%)					
Never	97 (30.7)	53 (52.5)	293 (50.0)	657 (33.8)	37 (17.1)
Previous smoker	185 (58.5)	36 (35.6)	255 (43.5)	1186 (61.1)	145 (67.1)
Current smoker	34 (10.8)	12 (11.9)	38 (6.5)	98 (5.0)	34 (15.7)
Alcohol use, N (%)					
Never or previous	55 (17.4)	46 (45.5)	55 (9.4)	1574 (29.6)	23 (10.6)
Current	261 (82.6)	55 (54.5)	531 (90.6)	1367 (70.4)	193 (89.4)
Sex					
Male	196 (62.0)	0 (0.0)	586 (100.0)	1234 (63.6)	173 (80.1)
Female	120 (38.0)	101 (100.0)	0 (0.0)	707 (36.4)	43 (19.9)
BMI, median (IQR)	26.37 (4.58)	26.84 (7.41)	26.25 (2.36)	25.83 (4.67)	26.11 (4.74)
Comorbidities, N (%)					
Zero	87 (27.5)	39 (38.6)	167 (28.5)	722 (37.2)	58 (26.9)
One	104 (32.9)	23 (22.8)	185 (31.6)	589 (30.3)	59 (27.3)
Two or more	125 (39.6)	39 (38.6)	234 (39.9)	630 (32.5)	99 (45.8)
Cancer type, N (%)					
Ovarian Cancer		63 (62.4)			
Endometrial Cancer		38 (37.6)			
Colorectal Cancer	316 (100.0)			1941 (100.0)	
Bladder Cancer					216 (100.0)
Prostate Cancer			586 (100.0)		
Cancer stage^2^, N (%)					
I	109 (34.5)	73 (72.3)	305 (52.0)	523 (26.9)	153 (70.8)
II	89 (28.2)	7 (6.9)	158 (27.0)	518 (26.7)	43 (19.9)
III	118 (37.3)	21 (20.8)	123 (21.0)	900 (46.4)	20 (9.3)
Time since diagnosis (in days), median (IQR)	20.00 (12.00)	73.05 (36.53)	40.00 (11.00)	32.00 (23.00)	42.03 (16.01)
Receiving treatment at T_baseline_ questionnaire, N (%)	12 (3.8)	42 (41.6)	60 (10.8)	561 (28.9)	131 (60.6)
Underwent systemic treatment, N (%)	94 (29.7)	31 (30.7)	1 (0.2)	247 (12.7)	14 (6.5)
Underwent radiotherapy, N (%)	41 (13.0)	21 (20.8)	205 (35.0)	414 (21.3)	22 (10.2)
Underwent surgery, N (%)	312 (98.7)	99 (98.0)	214 (36.5)	1,855 (95.6)	47 (21.8)
Quality of life, median (IQR)	83.33 (16.67)	75.00 (25.00)	83.01 (15.63)	83.33 (16.67)	83.33 (16.67)
Physical functioning (median, IQR)	100.00 (13.33)	86.67 (20.00)	100.00 (13.33)	100.00 (13.33)	93.33 (26.67)
Role functioning, median (IQR)	100.00 (16.67)	66.66 (33.33)	100.00 (16.67)	100.00 (33.33)	100.00 (33.33)
Emotional functioning, median (IQR)	83.33 (25.00)	83.33 (33.33)	91.67 (25.00)	83.33 (25.00)	83.33 (33.33)
Cognitive functioning, median (IQR)	100.00 (16.67)	83.33 (33.33)	100.00 (16.67)	100.00 (16.67)	100.00 (16.67)
Social functioning, median (IQR)	100.00 (16.67)	83.33 (33.33)	100.00 (0.00)	100.00 (33.33)	100.00 (16.67)
Fatigue, median (IQR)	11.11 (33.33)	33.33 (33.33)	0.00 (22.22)	22.22 (33.33)	22.22 (33.33)
Nausea/vomiting, median (IQR)	0.00 (0.00)	0.00 (0.00)	0.00 (0.00)	0.00 (0.00)	0.00 (0.00)
Pain, median (IQR)	0.00 (116.67)	16.67 (33.33)	0.00 (16.67)	0.00 (33.33)	16.67 (33.33)
Dyspnoea,, median (IQR)	0.00 (0.00)	0.00 (33.33)	0.00 (0.00)	0.00 (0.00)	0.00 (0.00)
Sleeping disturbance,, median (IQR)	20.50 (33.33)	33.33 (66.67)	0.00 (33.33)	0.00 (33.33)	0.00 (33.33)
Appetite loss, median (IQR)	0.00 (0.00)	0.00 (33.33)	0.00 (0.00)	0.00 (0.00)	0.00 (0.00)
Constipation, median (IQR)	0.00 (0.00)	0.00 (33.33)	0.00 (0.00)	0.00 (0.00)	0.00 (0.00)
Diarrhoea, median (IQR)	0.00 (33.33)	0.00 (0.00)	0.00 (0.00)	0.00 (33.33)	0.00 (0.00)
Financial difficulties, median (IQR)	0.00 (0.00)	0.00 (0.00)	0.00 (0.00)	0.00 (0.00)	0.00 (0.00)
Has clinically-relevant fatigue (T_endpoint_), N (%)	36 (11.4)	26 (25.7)	53 (9.0)	225 (11.6)	37 (17.1)

Overall, CRF was present in 13.2% of the patients at T_baseline_ and in 11.9% at T_endpoint_. The gynaecological cancer cohort had the largest proportion of patients with CRF at T_endpoint_ (N = 26; 25.7%), while the prostate cancer cohort had the smallest proportion (N = 53; 9.0%) (Table 1).

Of the 32 initial predictor variables, only cancer stage, systemic treatment, radiotherapy, and surgery were not statistically significantly associated with CRF at T_endpoint_ based on imputed data (Table 2).

**Table 2:** Univariable analysis of predictor variables at T_baseline_ with clinically-relevant fatigue at T_endpoint_

Predictor variable at T_baseline_	Clinically-relevant fatigue at T_endpoint_ (N = 377)	Not clinically-relevant fatigue at T_endpoint_ (N = 2,783)	P-value
Age at questionnaire, median (IQR)	67.00 (15.03)	67.06 (12.00)	< 0.01*
BMI, median (IQR)	26.81 (5.45)	25.95 (3.93)	< 0.01*
Days since diagnosis, median (IQR)	35.00 (22.03)	35.00 (22.00)	< 0.01*
Days between diagnosis and treatment start, median (IQR)	29.00 (18.00)	30.06 (21.00)	< 0.01*
Quality of life, median (IQR)	66.67 (25.00)	83.33 (25.00)	< 0.01*
Physical functioning, median (IQR)	80.00 (26.67)	100.00 (13.33)	< 0.01*
Role functioning, median (IQR)	66.67 (66.67)	100.00 (33.33)	< 0.01*
Emotional functioning, median (IQR)	75.00 (33.33)	83.33 (25.00)	< 0.01*
Cognitive functioning, median (IQR)	83.33 (33.33)	100.00 (16.67)	< 0.01*
Social functioning, median (IQR)	83.33 (33.33)	100.00 (16.67)	< 0.01*
Fatigue, median (IQR)	44.44 (44.44)	11.11 (33.33)	< 0.01*
Nausea/vomiting, median (IQR)	0.00 (16.67)	0.00 (0.00)	< 0.01*
Pain, median (IQR)	16.67 (50.00)	0.00 (16.67)	< 0.01*
Dyspnoea, median (IQR)	0.00 (33.33)	0.00 (0.00)	< 0.01*
Sleeping disturbance,, median (IQR)	33.33 (66.67)	0.00 (33.33)	< 0.01*
Appetite loss, median (IQR)	0.00 (33.33)	0.00 (0.00)	< 0.01*
Constipation, median (IQR)	0.00 (33.33)	0.00 (0.00)	< 0.01*
Diarrhoea, median (IQR)	0.00 (33.33)	0.00 (33.33)	< 0.01*
Financial difficulties, median (IQR)	0.00 (0.00)	0.00 (0.00)	< 0.01*
Education level, N (%)			< 0.01*
Lower/Primary	36 (9.5)	138 (4.9)	
Secondary (High school)	140 (37.1)	870 (31.3)	
Secondary (Vocational)	102 (27.1)	721 (25.9)	
Vocational/ University	99 (26.3)	1054 (37.9)	
Marital status, N (%)			< 0.01*
Married/ Cohabitating	268 (71.1)	2217 (79.7)	
Single/Divorced	109 (28.9)	566 (20.3)	
Smoking history, N (%)			< 0.01*
Never	110 (29.2)	1026 (36.9)	
Previous smoker	235 (62.3)	1573 (56.5)	
Current smoker	32 (8.5)	184 (6.6)	
Alcohol use, N (%)			< 0.01*
Yes	173 (45.9)	1372 (49.3)	
No	204 (54.1)	1411 (50.7)	
Sex			< 0.01*
Male	233 (61.8)	1956 (70.3)	
Female	144 (38.2)	827(29.7)	
Comorbidities, N (%)			< 0.01*
0	76 (20.2)	998 (35.9)	
1	103 (27.3)	856 (30.8)	
> 1	198 (52.5)	929 (33.4)	
Cancer type, N (%)			< 0.01*
Ovarian Cancer	13 (3.4)	50 (1.8)	
Endometrial Cancer	13 (3.4)	25 (0.9)	
Colorectal Cancer	261 (69.2)	1996 (71.7)	
Bladder Cancer	37 (9.8)	179 (6.4)	
Prostate Cancer	53 (14.1)	533 (19.2)	
Cancer stage, N (%)			0.60
I	131 (34.7)	1032 (37.1)	
II	97 (25.7)	718 (25.8)	
III	149 (39.5)	1033 (37.1)	
Currently undergoing treatment, N (%)			< 0.01*
Yes	119 (31.6)	687 (24.7)	
No	258 (69.4)	2,096 (76.3)	
Underwent systemic treatment, N (%)			0.08
Yes	57 (15.1)	330 (11.9)	
No	320 (84.9)	2453 (88.1)	
Underwent radiotherapy, N (%)			0.12
Yes	96 (25.5)	607 (21.8)	
No	281 (74.5)	2176 (78.2)	
Underwent surgery, N (%)			0.13
Yes	290 (76.9)	2237 (80.4)	
No	87 (23.1)	546 (19.6)	

Predictor values for patient data at T_baseline_ after imputation. P-value were reported following Wilcoxon rank-sum tests for continuous variables and chi-square tests for categorical variables. P-values were attained per imputed dataset and averaged. “*” indicated significance (p < 0.05)

### Predicting clinically relevant future fatigue at T_endpoint_

**Fig. 2:**
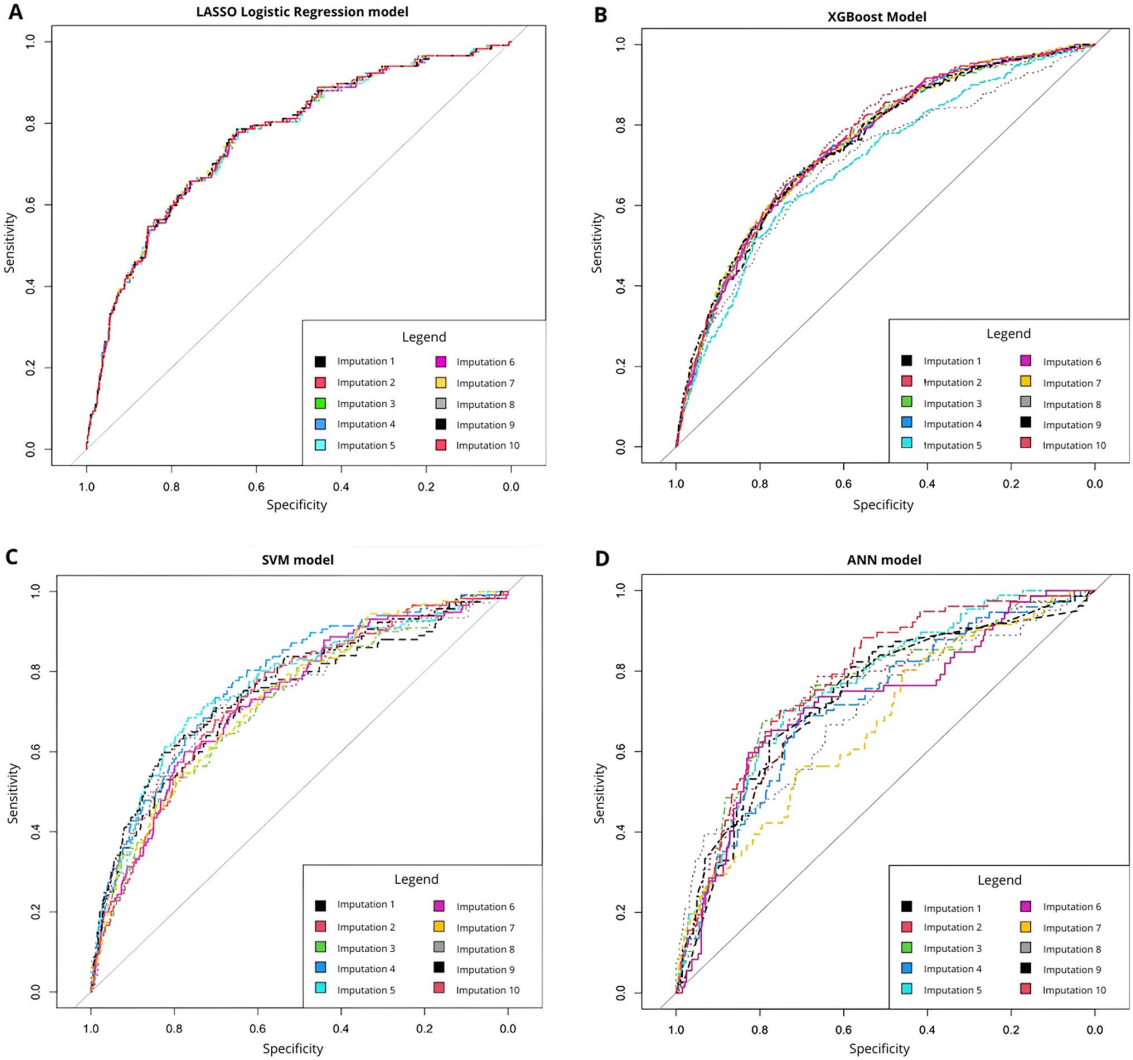
Receiver operating curves for each prediction model. Each imputed dataset was plotted and overlayed onto the overall chart

**Fig. 3:**
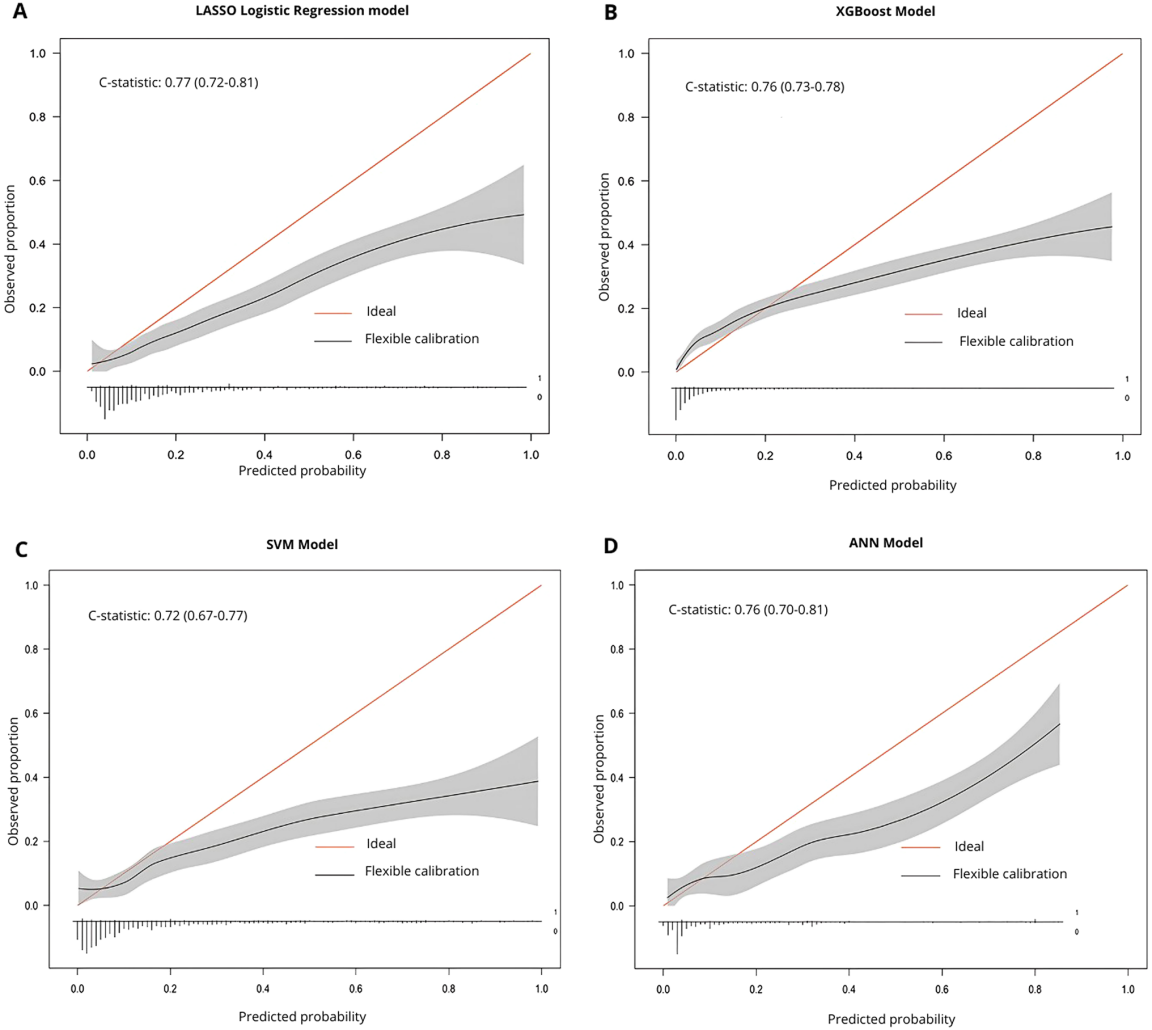
Calibration plots for each prediction model

The logistic regression model had the highest average balanced accuracy (0.653; SD = 0.023), precision (0.420; SD = 0.049), sensitivity (0.373; SD = 0.045), and C-statistic (0.769; SD = 0.024) (Table 3; Fig. 2). Thus, with respect to these metrics, the ML models did not outperform the logistic regression model, as initially hypothesized. Further, with respect to specificity, only the XGBoost model outperformed the logistic regression model (0.949; SD = 0.007). The XGBoost model had the lowest average balanced accuracy (0.585; SD = 0.014) and sensitivity (0.220; SD = 0.026), and the SVM model had the lowest average C-statistic (0.728; SD = 0.028). The logistic regression model had the highest average R-squared value (0.035; SD = 0.069) and the closest average calibration slope to the ideal (0.686; SD = 0.077) (Fig. 3). Finally, sensitivity analysis on the logistic regression model showed that model performance was best at the original CRF threshold (fatigue score >  = 40) (Appendix 5).

**Table 3:** Statistical output of each prediction model when predicting presence of future fatigue

Metric	Logistic Regression	XGBoost	SVM	ANN
Accuracy	0.861 (0.011)	0.863 (0.008)	0.838 (0.015)	0.825 (0.021)
Balanced Accuracy	0.652 (0.023)	0.585 (0.014)	0.627 (0.025)	0.622 (0.043)
Precision	0.420 (0.049)	0.366 (0.043)	0.322 (0.043)	0.308 (0.071)
Sensitivity	0.373 (0.045)	0.220 (0.026)	0.354 (0.049)	0.353 (0.026)
Specificity	0.929 (0.010)	0.949 (0.007)	0.904 (0.010)	0.891 (0.742)
F1-score	0.395 (0.044)	0.273 (0.030)	0.344 (0.053)	0.347 (0.061)
C-statistic	0.769 (0.024)	0.744 (0.024)	0.728 (0.028)	0.742 (0.034)
R-squared	0.035 (0.069)	-0.027 (0.135)	-0.148 (0.094)	-0.092 (0.137)
Calibration Slope	0.686 (0.077)	0.458 (0.080)	0.489 (0.067)	0.553 (0.139)

Standard deviations are shown in brackets

### Internal–external cross-validation

Internal–external cross-validation was conducted on the logistic regression model after it was deemed to perform better than the other prediction models (Table 4). When the prostate cancer cohort was used as the validation population and other cohorts as development population, external validation attained the highest average accuracy (0.913; SD = 0.003), precision (0.541; SD = 0.038), and specificity (0.979; SD = 0.003). However, the prostate cancer cohort had the lowest average balanced accuracy (0.611; SD = 0.007) and sensitivity (0.243; SD = 0.014). When the colorectal cancer (PROCORE) cohort was used as the validation dataset, it attained the highest average balanced accuracy (0.693; SD = 0.007), sensitivity (0.511; SD = 0.014), and C-statistic (0.818; SD = 0.001). The bladder cancer cohort as the validation dataset had the lowest average C-statistic (0.736; SD = 0.004).

**Table 4:** Statistical output of each cohort during internal–external validation

Metric	Logistic Regression Output	Bladder Cancer	Colorectal Cancer	Prostate Cancer—PROCORE	Prostate Cancer—ProZib	Gynaecological cancer
Accuracy	0.861 (0.011)	0.796 (0.003)	0.851 (< 0.001)	0.836 (0.002)	0.913 (0.003)	0.757 (0.005)
Balanced Accuracy	0.652 (0.023)	0.630 (0.002)	0.619 (0.002)	0.693 (0.007)	0.611 (0.007)	0.665 (0.009)
Precision	0.420 (0.049)	0.399 (0.008)	0.345 (0.001)	0.345 (0.006)	0.541 (0.038)	0.532 (0.011)
Sensitivity	0.373 (0.045)	0.378 (< 0.001)	0.316 (0.004)	0.511 (0.014)	0.243 (0.014)	0.2473 (0.019)
Specificity	0.929 (0.010)	0.882 (0.004)	0.921 (0.001)	0.875 (< 0.001)	0.979 (0.003)	0.856 (0.006)
F1-score	0.395 (0.044)	0.371 (0.002)	0.329 (0.003)	0.400 (0.002)	0.347 (0.011)	0.487 (0.017)
C-statistic	0.769 (0.024)	0.776 (0.001)	0.745 (0.001)	0.818 (0.001)	0.787 (0.003)	0.736 (0.004)

Each metric was calculated per imputed dataset and averaged. Standard deviations are shown in brackets

**Fig. 4:**
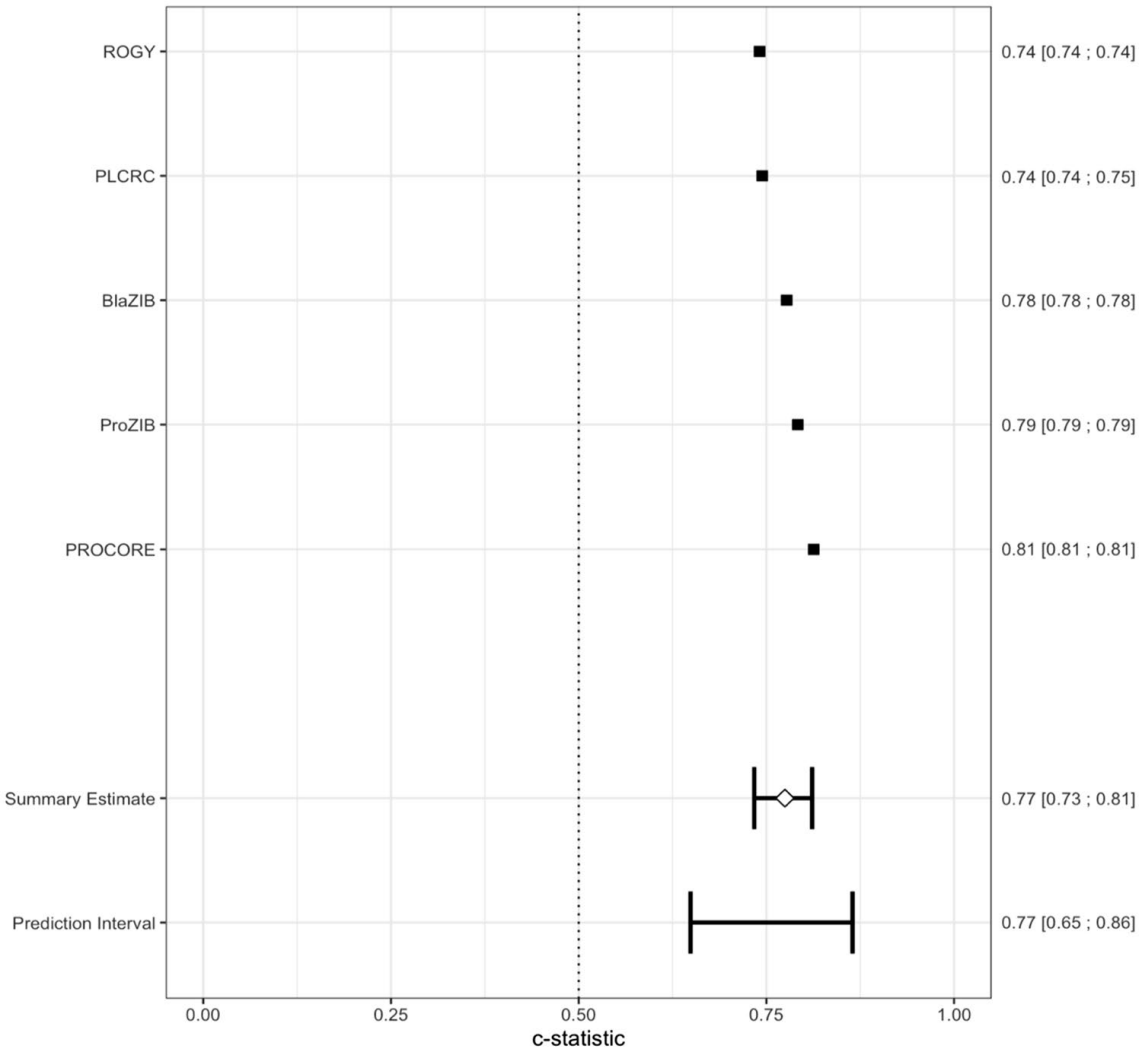
Forest plot from internal–external validation meta-analysis

A meta-analysis was conducted of all C-statistics in each internal–external validation iteration. The pooled C-statistic was 0.77 (95%CI 0.73–0.81), indicting that, relative to each other, each cohort was able to consistently discriminate CRF (Fig. 4). Similar results were shown from a sensitivity analysis based on cancer type (Appendix 6). In the final overall model, highest significant beta coefficients were for education status, smoking history, number of comorbidities, physical activity, fatigue, cancer type (ovarian cancer) and cancer stage (Appendix 7). Education status, smoking history, number of comorbidities, fatigue score and cancer stage were also consistently the highest (top 5) beta coefficients across individual cohort studies’ models (not tabulated).

## Discussion

This study aimed to develop and compare prediction models to predict CRF in different, cancer patient cohorts. The goal was set to address a research gap and support clinicians in selecting patients who need long-term fatigue-related supportive care. This study found that the logistic regression model with LASSO regularisation performed better than the ML-based models, indicating that a simpler model is preferred for creating prediction models for future CRF. The combination of multiple, heterogenous cohorts with different cancer types in the same model was feasible, as the logistic regression model remained stable across the various cohort studies. However, for all the models, calibration was poor, which suggested a high risk of underdiagnosing CRF. This poor calibration may be explained by class imbalance, i.e., the lack of patients with CRF as an outcome.

Unfortunately, SMOTE only partially improved the class imbalance in our study. The influence of class imbalance was reflected in an imbalance between sensitivity and specificity, with sensitivity being near zero. In a clinical application, it is preferable to have both high sensitivity and high specificity, as this ensures that type I (false positives) and type II errors (false negatives) are minimised [51]. Although we based our dichotomisation on the threshold for clinical importance defined by Giesinger et al. [10], a lower threshold for fatigue did not significantly reduce class imbalance in our sensitivity analysis. Therefore, using a continuous representation of the fatigue scales as an outcome measure may potentially alleviate class imbalance.

Following internal–external cross-validation, each cohort performed consistently with regard to each other as well as to the overall model with all cohorts included. This was evident in most of the performance measures. Regarding balanced accuracy, the average value was consistent throughout all cohorts, albeit still as low, as the overall model. This meant that, when tested, each cohort performed similarly with respect to class imbalance [48]. Aside from balanced accuracy, this consistency was also observed in each cohort’s sensitivity and specificity.

A strength of this study is the combination of multiple, heterogenous cohorts with different cancer types in the same model. Further, this study demonstrates that predictors missed in previous studies [18, 19], such as functioning and symptoms, can be included into a prediction model for CRF. Alternatively, other questionnaires measuring these constructs could be used in future research. Limitations include the initial data collection and processing, which revealed a considerable class imbalance between patients with and without CRF at both T_baseline_ and T_endpoint._ Previous studies have highlighted the issue of non-respondents and attrition bias within the PROFILES registry [53, 54], indicating that patients with lower quality of life and more symptoms are underrepresented in the current study. Hence, a selection bias favouring less-fatigued patients in our study is inevitable. This bias was reflected in the relatively low proportion of patients reporting CRF (11.9%), which is much lower than other studies studying CRF (e.g., 38% [18]). Therefore, while SMOTE could not completely correct the class imbalance in our study, and sensitivity analysis using different thresholds did not suggest an improvement, future studies should focus on reducing selection and attrition bias. Moreover, when dealing with class imbalance, future research could also investigate whether using other methods, e.g., simple random oversampling, provides comparable performance to SMOTE within this context.

Due to the current unavailability of new, unseen data, external validation was not possible for this study. We believe that the internal–external validation procedure was a robust method for assessing the model’s performance across different cohorts, offering preliminary evidence for external validity. Future research should utilize new datasets from the PROFILES registry, including additional cancer types, as they become available. Furthermore, collaborations with external and international institutions will be essential for conducting comprehensive external validation across diverse cancer populations. Subgroup analyses should also be conducted to evaluate the model’s performance across various demographic and clinical subgroups. Additionally, the exploration of federated learning techniques is recommended to enable model training across multiple centers while ensuring the protection of patient data privacy.

Further, there is value in investigating the extent to which a patient’s fatigue changes over the shorter term (e.g., at 12 months) or within intervals (e.g., every three months). Output from such models could help to adapt care more quickly [55], also when predicting other symptoms. Moreover, the timepoints defined in the current study provided nuanced differences for prediction and subsequent interpretation. Future research should, therefore, incorporate timeline anchors (e.g., date of diagnosis) into prediction models to provide a well-defined point of reference is available for clinical interpretation.

In addition, given that CRF can potentially emerge at any timepoint after baseline measurements, a longitudinal time-series model should be explored as a modelling strategy to capture CRF. In our analyses, we developed statistical models for predicting the (non-)presence of CRF 12 months after diagnosis. Due to insufficient time-series data, we could not adopt a longitudinal modeling approach. The main benefit of a longitudinal time-series is that it can capture CRF at any time point since diagnosis and is not restricted to one year after diagnosis. However, the main disadvantage is that longitudinal models are more complex and, therefore, more challenging to interpret. We expect that time-series models would better capture future fatigue because they utilize not only baseline fatigue as input but the whole time-series, which can include autocorrelation and may be informative for future predictions (i.e., if a person has elevated fatigue in month X, they are likely to experience fatigue in month X + 1 as well). A potential challenge is the consistent availability of the data at evenly spaced time points. This condition is not always met for different reasons and can create heteroscedasticity in the data (i.e., unevenly distributed observations across time), which would need to be addressed by a more complex statistical model, that accounts for the (un)conditional variance, such as weighted least squares, which uses the inverse of the (estimated) variance of each observation as a weight.

Finally, data on recurrence and its subsequent treatment were not incorporated in the current study, which could have had a substantial effect on the occurrence of CRF at T_endpoint_. Recurrence and its related treatment regimen could potentially be incorporated into the model in the form of time-varying parameters. However, this would require including “future” data not observed at T_baseline_, which was not the purpose of the current model. Future research could use a counterfactual interpretation of the predictions of such a time-varying parameter model by, for example, predicting fatigue at T_endpoint_ conditional on the presence (or absence) of recurrence at a future time point.

In this study, we did not discuss ML explainability measures that have received prominence in recent years, such as Shapley values, because our best model (i.e., logistic regression) is readily explainable: a linear relationship is established between CRF and the predictors in the logit space. The coefficients indicate the change of CRF per unit change of the predictors, and, therefore, clinicians can directly see the effect of each predictor on CRF. Had any of the more complex ML models shown better performance, ML explainability would be paramount to investigate how the models arrive at their predictions.

To conclude, this study demonstrated that CRF prediction modelling using logistic regression and ML is feasible, when combining multiple, heterogenous cohorts. Although the model’s discrimination was good, the low sensitivity and poor calibration indicated a high likelihood of underdiagnosis of future CRF. Yet, compared to different ML algorithms, logistic regression performed better and was robust across cohorts, which suggests the advantage of having a simpler model to predict CRF. Despite demonstrating how CRF can be predicted, the clinical applicability of the model based on this study remains inconclusive. Therefore, to support clinicians in selecting patients who need long-term fatigue-related supportive care, future research should consider reducing selection/attrition bias and include recurrence data when developing CRF-related prediction models.

## Supplementary Information

Below is the link to the electronic supplementary material.

Supplementary file1 (DOCX 183 kb)

## Data Availability

The data that support the findings of this study are available from each cohort studies’ principal investigator (NE; FM; KA; MK). Restrictions apply to the availability of these data, which were used under license for this study. Data are available through the corresponding author with the permission of the respective study principal investigators. The code of our analyses can be accessed through GitHub (https://github.com/IKNL/prediction_modeling_fatigue).
