# Supplementary material for: Development of a prediction model for clinically-relevant fatigue: a multi-cancer approach
Source: Qual Life Res. 2024 Nov 9;34(1):231–45. doi: 10.1007/s11136-024-03807-9 (PMC11802682; doi:10.1007/s11136-024-03807-9)
Supplement: Supplementary file 1 — Supplementary file1 (DOCX 183 kb) [file 11136_2024_3807_MOESM1_ESM.docx]

Appendix 1: Overview of data collection time intervals for each cohort study.

| **Cancer/Cohort** | **Time since diagnosis**  **(months)** | | | | | | | **Time since initial treatment (months)** | | |
| --- | --- | --- | --- | --- | --- | --- | --- | --- | --- | --- |
|  | 0 | 3 | 6 | 12 | 18 | 24 | 36 | 0 | 12 | 24 |
| Prostate Cancer - PROCORE | X |  |  | X |  | X |  |  |  |  |
| Gynaecological Cancer |  |  |  |  |  |  |  | X | X | X |
| Bladder Cancer | X |  | X | X |  | X |  |  |  |  |
| Prostate Cancer - ProZIB | X |  |  | X |  | X |  |  |  |  |
| Colorectal Cancer | X | X | X | X | X | X |  |  |  |  |

Appendix 2: Variables pre-processed during analysis (set to null/missing)

| **Predictor Variable** | **Criterion** | **Reasoning** |
| --- | --- | --- |
| Patient’s BMI at T_baseline_ questionnaire | BMI > 251.1 | Exceeds highest recorded BMI |
|  | BMI < 6.7 | Exceeds lowest recorded BMI |
| Time between diagnosis and T_baseline_ questionnaire | Days < 0 | Patient’s data is outside the study’s timeframe |
| Age at T_baseline_ questionnaire | Years > 122 | Exceeds highest recorded age |
| EORTC QLQ-C30 social functioning score | Score > 100 | Coding error |
| EORTC QLQ C30 financial difficulty score | Score > 100 | Coding error |

Appendix 3. Study methodology flowchart.


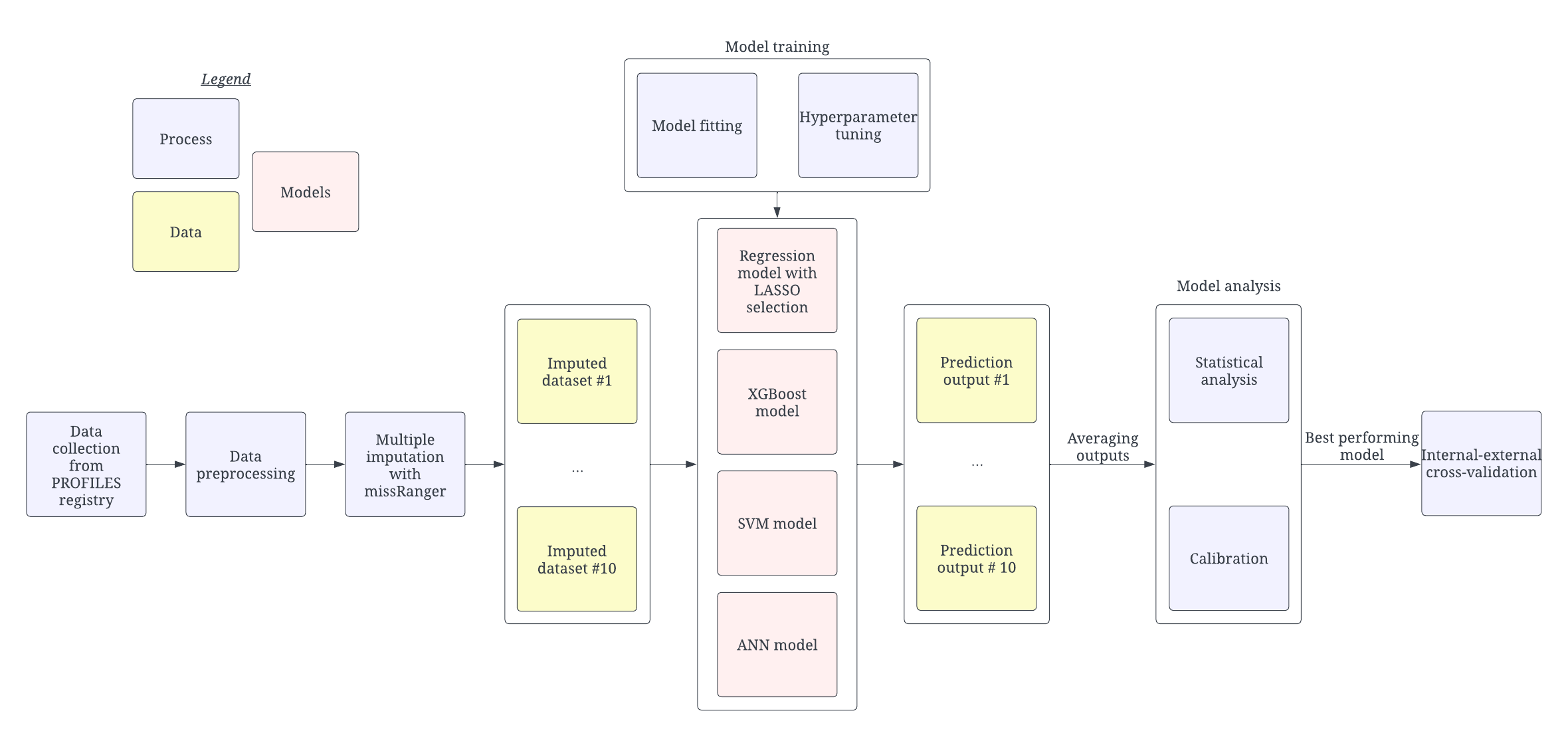


Appendix 4: Hyperparameters tuned for each prediction model

| **Model** | **Hyperparameters tuned** | **Range** |
| --- | --- | --- |
| Logistic regression | Alpha constraint | [0.01,10^10^] |
| XGBoost | Maximum tree depth | [3,7] |
|  | Step size of each boosting step (shrinkage) | [0.01,1) |
|  | # of boosting iterations | (200,500) |
|  | Minimum loss reduction | (0.1,10) |
|  | Subsample ratio of columns | [0.5,0.7] |
|  | Minimum sum of instance weight | - |
|  | Subsample percentage | - |
| SVM | Cost – “C” constant in Lagrange formulation | [0.01,10] |
| ANN | Number of hidden units in the network | [5,8] |
|  | Weight decay | [0.01,1] |

Appendix 5: Sensitivity analysis results (applied to logistic regression model with LASSO selection)

| **Metric** | **Fatigue score >= 39 (Reference)** | **Fatigue score >= 20** | **Fatigue score >= 30** | **Fatigue score >= 50** | **Fatigue score >= 60** |
| --- | --- | --- | --- | --- | --- |
| Accuracy | 0.861  (0.011) | 0.636  (0.016) | 0.763  (0.015) | 0.870  (0.011) | 0.895  (0.010) |
| Balanced Accuracy | 0.652  (0.023) | 0.607  (0.012) | 0.616  (0.016) | 0.620  (0.029) | 0.646  (0.040) |
| Precision | 0.420  (0.049) | 0.770  (0.034) | 0.620  (0.046) | 0.252  (0.045) | 0.180  (0.042) |
| Sensitivity | 0.373  (0.045) | 0.282  (0.022) | 0.300  (0.029) | 0.322  (0.052) | 0.368  (0.073) |
| Specificity | 0.929  (0.010) | 0.931  (0.011) | 0.933  (0.010) | 0.918  (0.010) | 0.919  (0.009) |
| F1-score | 0.395  (0.044) | 0.412  (0.026) | 0.404  (0.032) | 0.282  (0.045) | 0.242  (0.050) |
| C-statistic | 0.769  (0.024) | 0.723  (0.017) | 0.785  (0.014) | 0.767  (0.026) | 0.778  (0.035) |
| R-squared | 0.035  (0.069) | -0.134  (0.073) | 0.148  (0.047) | -0.223  (0.104) | -0.749  (0.222) |
| Calibration Slope | 0.686  (0.077) | 0.644  (0.061) | 0.662  (0.065) | 0.641  (0.080) | 0.718  (0.118) |

*Each metric was calculated per imputed dataset and averaged. Standard deviations are shown in brackets.*

Appendix 6: Sensitivity analysis internal-external validation by cancer type. Meta analysis forest plot based on cancer type.

| **Metric** | **Logistic Regression Output** | **Bladder Cancer** | **Colorectal Cancer** | **Gynaecological Cancer** | **Prostate Cancer** |
| --- | --- | --- | --- | --- | --- |
| Accuracy | 0.861  (0.011) | 0.797  (0.001) | 0.848  (0.001) | 0.763  (0.016) | 0.913  (0.001) |
| Balanced Accuracy | 0.652  (0.023) | 0.620  (0.001) | 0.627  (0.002) | 0.575  (0.021) | 0.617  (0.004) |
| Precision | 0.420  (0.049) | 0.395  (0.004) | 0.343  (0.002) | 0.390  (0.053) | 0.543  (0.021) |
| Sensitivity | 0.373  (0.045) | 0.351  (<0.001) | 0.340  (0.003) | 0.254  (0.037) | 0.255  (0.019) |
| Specificity | 0.929  (0.010) | 0.889  (0.002) | 0.915  (<0.001) | 0.896  (0.016) | 0.979  (0.010) |
| F1-score | 0.395  (0.044) | 0.371  (0.002) | 0.329  (0.003) | 0.487  (0.017) | 0.341  (0.003) |
| C-statistic | 0.769  (0.024) | 0.777  (0.001) | 0.752  (0.001) | 0.673  (0.007) | 0.792  (0.001) |

*Each metric was calculated per imputed dataset and averaged. Standard deviations are shown in brackets.*


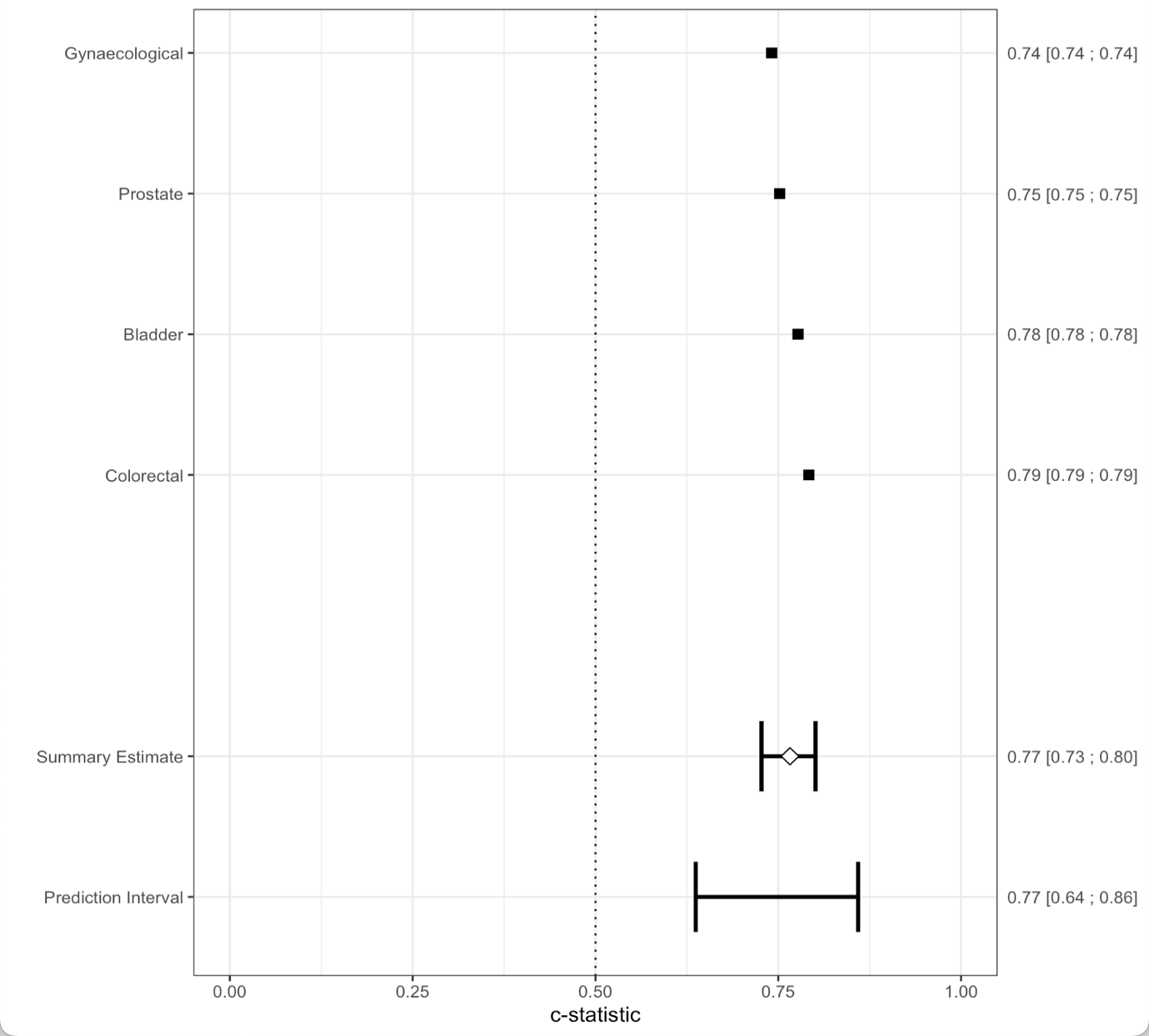


Appendix 7: Beta coefficients for the final logistic regression model.

| **Predictor** | **Beta Coefficient (SD)** |
| --- | --- |
| (Intercept) | -0.348 (0.049) |
| Education level (primary education) | 0.857 (0.010) |
| Education level (higher education) | -0.040 (0.025) |
| Marital status (married/cohabitating) | 0.336 (0.005) |
| Smoking history (never smoked) | -0.288 (0.028) |
| Smoking history (current smoker) | 0.185 (0.011) |
| Alcohol use (no) | 0.005 (0.013) |
| Comorbidities (zero) | -0.143 (0.015) |
| Comorbidities (one) | 0.086 (0.015) |
| Quality of life | -0.001 (<0.001) |
| Physical functioning | -0.047 (<0.001) |
| Social functioning | 0.009 (<0.001) |
| Fatigue | 0.034 (<0.001) |
| Nausea/vomiting | -0.005 (<0.001) |
| Dyspnoea | 0.002 (<0.001) |
| Appetite loss | -0.011 (<0.001) |
| Diarrhoea | -0.008 (<0.001) |
| Financial difficulties | 0.016 (<0.001) |
| Cancer type (Ovarian cancer) | 0.267 (0.009) |
| Cancer stage (II) | 0.275 (0.002) |
| Cancer stage (III) | -0.172 (0.003) |
| BMI | 0.006 (<0.001) |
| Underwent radiotherapy (No) | 0.211 (0.003) |
| Underwent systemic therapy (No) | -0.818 (0.014) |
| Underwent systemic therapy (Yes) | 0.008 (0.013) |
| Age at questionnaire | -0.014 (<0.001) |
| Days since diagnosis | -0.026 (<0.001) |
| Currently under treatment (No) | 0.037 (0.002) |

Note: Standard deviations (SD) report the variation of each predictor’s beta coefficient between imputed datasets and are shown in brackets.

Appendix 6 shows the beta coefficients of the predictors used in the final logistic regression model. After LASSO selection, 12 continuous predictors and 10 categorical predictors were included in the final model. Since one-hot encoding was used to model categorical predictors, categorical predictor levels with non-zero beta coefficients were included in the model. Note that because the displayed figures are an average between imputed datasets, for binary categorical variables, one level’s beta coefficient might not necessarily imply the inverse of another.

Of the included categorical predictors, low/primary educational level at had the highest beta coefficient (0.882; SD = 0.020) while no current alcohol use had the lowest beta coefficient (-0.353; SD = 0.011). Meanwhile, of the included continuous predictors, fatigue at T_baseline_ had the highest beta coefficient (0.034; SD = <0.001) while physical functioning at T_baseline_ had the lowest beta coefficient (-0.047; SD = <0.001).
